# Bibliometric analysis combined with visualization on universal trends and hot topics of LOX family in human diseases: 1995 to 2025

**DOI:** 10.3389/fonc.2025.1601261

**Published:** 2025-06-30

**Authors:** Tingting Yang, Heng Li, Wenxuan Zhou, Ning Zhang, Zhenyu Tian, Heming Wang, Yuyan Feng, Yunguang Chen, Zhen Wang

**Affiliations:** ^1^ Wuxi School of Medicine, Jiangnan University, Wuxi, China; ^2^ Department of Nuclear Medicine, Affiliated Hospital of Jiangnan University, Wuxi, China

**Keywords:** bibliometric analysis, lysyl oxidase, diseases, fibrosis, cancer, extracellular matrix

## Abstract

**Introduction:**

Lysyl oxidase (LOX) is crucial for modifying collagen and elastin, thereby preserving tissue integrity. Aberrant LOX activity has been associated with a multitude of health disorders, including cutaneous, pulmonary, fibrotic, cardiovascular diseases, and cancer. In cancer, LOX can either promote or inhibit tumor development, and its expression level is closely correlated with patient prognosis.

**Methods:**

This research utilized data retrieved from the Web of Science Core Collection on May 30, 2025. The search strategies were crafted to target LOX – related terms while excluding irrelevant ones, and the data were limited to English – language articles. Over the past 30 years, 9261 LOX – related publications were identified. The number of articles exhibited an upward trend, especially in the past decade. The United States, China, and Japan were the leading countries in terms of publication output, with institutions like Harvard University and Boston University being highly productive.

**Results:**

This research utilized data retrieved from the Web of Science Core Collection on May 30, 2025. The search strategies were crafted to target LOX – related terms while excluding irrelevant ones, and the data were limited to English – language articles. Over the past 30 years, 9261 LOX – related publications were identified. The number of articles exhibited an upward trend, especially in the past decade. The United States, China, and Japan were the leading countries in terms of publication output, with institutions like Harvard University and Boston University being highly productive.

**Discussion:**

This study presents an overview of LOX - related research. Comprehending the mechanisms of LOX can offer valuable perspectives on tumor biology. Future research on LOX – extracellular matrix interactions and associated gene pathways may lead to the development of novel diagnostic and treatment modalities targeting LOX.

## Introduction

The Lysyl oxidase (LOX) family of enzymes plays indispensable roles in the cross-linking and remodeling of the extracellular matrix (ECM), and participates in the post-translational modification of collagen and elastin (1). In mammals, the LOX family typically consists of four or five members: LOX, LOXL1, LOXL2, LOXL3, and LOXL4, depending on the nomenclature differences in the literature regarding LOX and LOXL0. Structurally, they are made up of three separate domains: an amino-terminal signal peptide that facilitates its secretion, a central catalytic domain that performs the oxidative deamination of lysine residues in collagens, and a carboxy-terminal regulatory domain (2–6). They share common features such as dependence on copper ions (Cu^2+^) and vitamin C as cofactors to catalyze the oxidation of lysine/hydroxylysine to aldehyde for ECM cross-linking. This unique structure enables LOX family to act as a critical regulator of tissue integrity, facilitating the formation of cross - linked collagen fibers that are essential for skin elasticity, vessel stability, and the strength of connective tissues (7–10)However, they differ in substrate preference, cellular signaling regulation, and expression during developmental stages. LOX primarily acts on collagen and elastin, while LOXLs can target a broader range of matrix proteins. LOXLs, especially LOXL2, can regulate signaling pathways in an enzyme-independent manner, whereas LOX function is more dependent on enzymatic activity. LOX is continuously expressed in adult tissues, while LOXLs are more active during embryonic development or tissue repair. Based on the characteristics of each LOX family member, we have compiled and described the LOX family members according to their molecular weight, primary tissue/cell expression, and core functions in **Table 1**.

**Table 1:** Function of each LOX family member.

Gene Name	Alias	Primary Expressing Tissues/Cells	Core FunctionLOX
LOX/LOXL0 (1, 115, 116)	LOX	Fibroblasts, endothelial cells, etc.	A classical lysyl oxidase that catalyzes the cross-linking of collagen and elastin to maintain ECM stability.
LOXL1 (1, 117, 118)	LOX-like 1	Skin, lungs, mammary glands, etc.	Involved in ECM cross-linking, regulating tissue fibrosis and tumor metastasis, including inhibiting breast cancer cell migration.
LOXL2 (1, 112, 119)	LOX-like 2	Tumor cells, fibroblasts	Promotes tumor angiogenesis, invasion, and metastasis, highly expressed in lung and ovarian cancers, associated with fibrosis.
LOXL3 (120, 121)	LOX-like 3	Embryonic tissues, placenta, skin	Participates in embryonic development and skin homeostasis, potentially related to hair development.
LOXL4 (122–124)	LOX-like 4	Kidneys, placenta, digestive tract	Regulates kidney development and injury repair, potentially involved in ECM remodeling and fibrotic processes.

LOX family of enzymes activity is crucial for preserving the structural integrity and mechanical properties of tissues like the skin, lungs, blood vessels, and other connective tissues (3–6, 8, 11). LOX facilitates the oxidation of lysine residues in proteins, leading to the formation of covalent cross-links essential for tissue elasticity and resilience (12). However, abnormal LOX activity has been associated with a broad spectrum of pathological conditions. For example, deficiencies in LOX or its isoforms are associated with severe cutaneous disorders like cutis laxa, characterized by skin fragility and reduced dermal elasticity due to impaired elastic fiber formation (13, 14). Similarly, LOX dysfunction is linked to pulmonary diseases, including alveolar wall destruction and impaired lung function, highlighting its importance in maintaining respiratory tissue architecture (15, 16). Moreover, altered LOX activity is involved in fibrotic diseases such as liver cirrhosis and pulmonary fibrosis, where excessive extracellular matrix (ECM) remodeling leads to tissue stiffening and organ dysfunction (17, 18). In addition to its role in connective tissues, LOX has attracted attention for its involvement in cardiovascular pathologies (19–21). Elastic fiber abnormalities caused by LOX deficiency can lead to vascular complications, such as aneurysms, emphasizing the enzyme’s critical function in maintaining vascular integrity (22–25).

Beyond these well - established roles, studies have also explored the potential of LOX in cancer biology (26). Emerging evidence suggests that LOX activity may contribute to tumor progression and metastasis by facilitating ECM remodeling, promoting cancer cell invasion, and enhancing angiogenesis (27, 28). Elevated LOX expression has been observed in various cancers, where it promotes ECM stiffening and the formation of a pro - tumorigenic niche (29, 30). Additionally, LOX activity triggers downstream signaling pathways like TGF-β and MAPK, which are strongly linked to epithelial-mesenchymal transition (EMT) and the preservation of cancer stem cells (31–33). LOX has also been shown to interact with key signaling pathways like focal adhesion kinase (FAK) - YAP/TAZ, which are crucial for tumor cell survival and proliferation (32, 34). Further clinical investigation into hepatocellular carcinoma (HCC) tissues revealed that HIF-1α is implicated in LOXL2 protein expression, correlates with poor prognostic factors, and drives processes such as EMT, migration, invasion, and vasculogenic mimicry in HCC cells by regulating LOXL2 (35). Additionally, lysyl oxidase (LOX) released from hypoxic breast tumor cells accumulates in premetastatic niches, cross-links type IV collagen within the basement membrane, and critically contributes to the recruitment of CD11b+ myeloid cells (36).In most cases, LOX is a pivotal factor in the restructuring of the tumor-related extracellular matrix, metastasis, and the premetastatic niche. These findings indicate that the LOX family could be a viable target for preventing and treating metastatic conditions. Nevertheless, LOX can also display antitumor effects in certain cancers, with its metabolic by-products potentially counteracting oncogenesis. For example, the expression levels of LOX are closely related to gastric cancer patient prognosis, with low expression usually associated with poorer clinical outcomes (37). LOX expression is downregulated in epidermal tumor cells of carcinomas but upregulated in the stroma adjacent to invasive tumor cells, and also they proved that LOX enzymatic activity is crucial for preserving the integrity of the dermis as well as maintaining the epidermal homeostasis but not for cancer progression (38). LOX mRNA is translated into an inactive LOX proprotein (pro-LOX) within the cytoplasm. Subsequently, pro-LOX is secreted from the cell, where the N-terminus of the protein is cleaved by BMP-1, resulting in the LOX propeptide (LOX-PP) and the mature, active LOX protein (39). Another study showed that 18-kDa propeptide domain of LOX (LOX-PP) from LOX strongly suppresses the invasive characteristics of lung and pancreatic cancer cells by targeting Bcl2 and NF-kappaB (40). Furtherly, LOX-PP expression was found to diminish the proliferative, migratory, anchorage-independent growth, and tumorigenic abilities of Ewing sarcoma in immunocompromised mice, while the C-terminal of LOX enzymatically active domain of LOX exerted opposing effects, indicating that LOX’s tumor-suppressive role is specifically attributable to its propeptide domain (41). This implies that treatments targeting LOX should be selective, targeting only those cancers where LOX promotes progression, potentially necessitating molecular diagnostic tools, and that LOX or its by-products could potentially serve as anticancer agents (10, 42, 43). Through an extensive literature review, this study aims to systematically analyze the current research status of LOX family and propose innovative and feasible future research directions by integrating it with hot topics in fibrotic diseases and cancer research. This will enhance comprehension of the mechanisms behind LOX’s involvement in tumor growth and advancement, offering fresh perspectives and possible targets for clinical treatment.

## Materials and methods

### Data acquisition and processing

The data employed for this bibliometric study were acquired from the Web of Science Core Collection (WoSCC) database, Scopus, PubMed, and Embase, incorporating the Science Citation Index Extended (SCIE), Social Science Citation Index (SSCI), and Emerging Sources Citation Index (ESCI). The information was obtained from the, Scopus, PubMed, and Embase from 1995 to 2025 on May 30, 2025. The search strategies were as follows:

1#. TS=“lysyl oxidase” OR “LOX” OR “LOXL1” OR “LOXL2” OR “LOXL3” OR “LOXL4” OR “lysyl oxidase like 1” OR “lysyl oxidase like 2” OR “lysyl oxidase like 3” OR “lysyl oxidase like 4”OR “Protein - lysine 6 - oxidase” OR “Copper - dependent amine oxidase”NOT TS=(“lipoxygenase” OR “lactate oxidase” OR “liquid oxygen”)

2#. TS=“lysyl oxidase” OR “LOX” OR “LOXL1” OR “LOXL2” OR “LOXL3” OR “LOXL4” OR “lysyl oxidase like 1” OR “lysyl oxidase like 2” OR “lysyl oxidase like 3” OR “lysyl oxidase like 4”OR “Protein - lysine 6 - oxidase” OR “Copper - dependent amine oxidase” AND TS=(“fibrosis” OR “fibrotic” OR “fibrogenesis”) NOT TS=(“lipoxygenase” OR “lactate oxidase” OR “liquid oxygen”)

3#. TS=“lysyl oxidase” OR “LOX” OR “LOXL1” OR “LOXL2” OR “LOXL3” OR “LOXL4” OR “lysyl oxidase like 1” OR “lysyl oxidase like 2” OR “lysyl oxidase like 3” OR “lysyl oxidase like 4”OR “Protein - lysine 6 - oxidase” OR “Copper - dependent amine oxidase”AND TS=(“cancer” OR “tumor” OR “neoplasm” OR “malignancy” OR “carcinoma”) NOT TS=(“lipoxygenase” OR “lactate oxidase” OR “liquid oxygen”)

**Figure 1:**
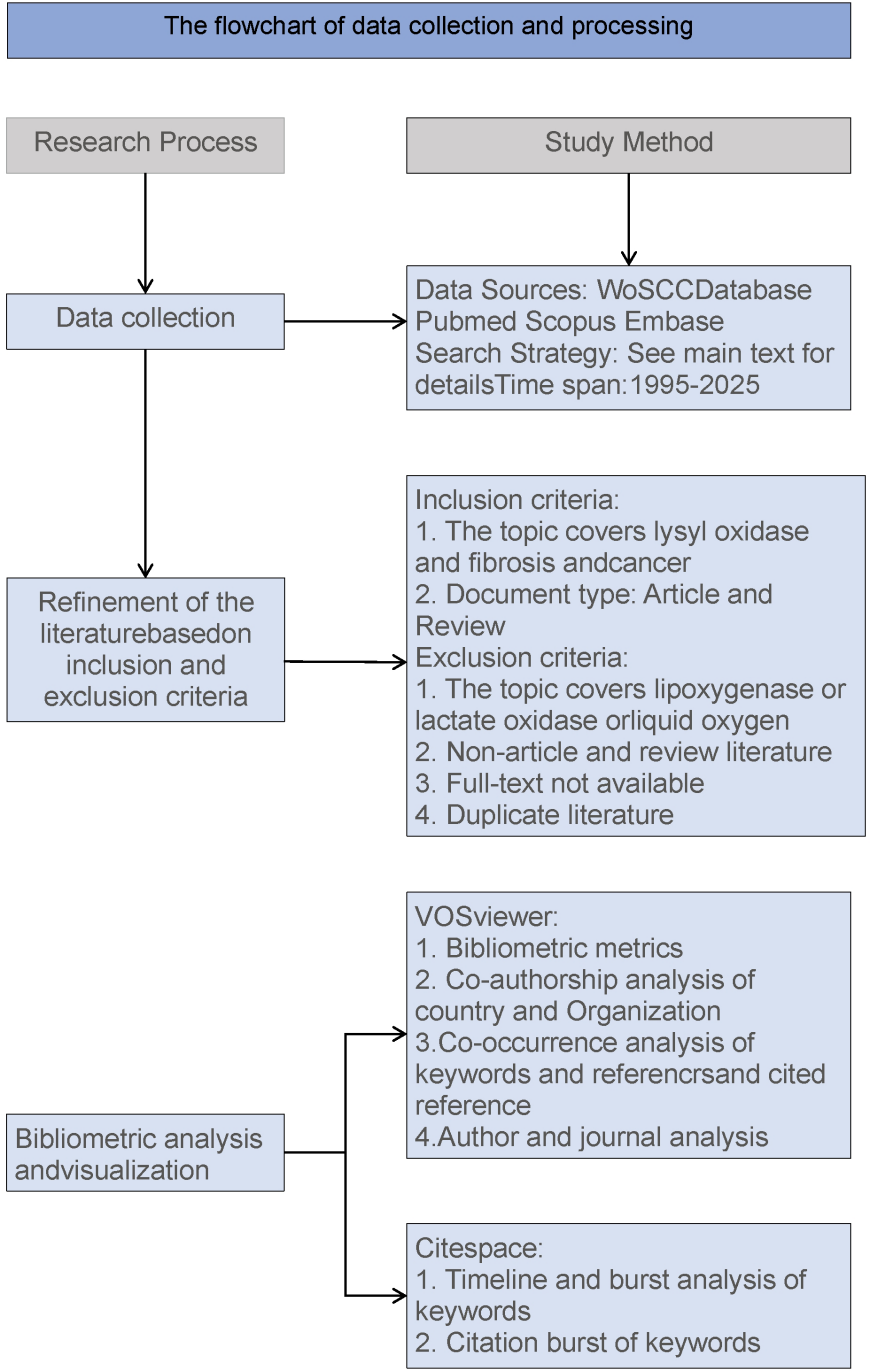
The flowchart of data collection and progressing.

The data were limited to English - language articles. After the above - mentioned selection process, a total of 8255 for 1# search strategy, 831 for 2# search strategy and 2041 for 3# search strategy papers according to the above strategies were obtained for bibliometric analysis (**Figure 1**).

### Bibliometric visualized analysis

VOSviewer (version 1.6.20; https://www.vosviewer.com/) was employed to conduct analyses of countries, organizations, journals, authors, co - cited authors, reference co - occurrences, and keyword co-occurrences (44). CiteSpace (version 6.3.R1; https://citespace.podia.com/) was used to analyze citation burst frequencies, keyword bursts (45). Additionally, Graphpad Prism 8 (https://www.graphpad.com/) was applied to analyze thosepublications on LOX from 1995 to 30 May, 2025.

## Results

### General literature trends on LOX from 1995 - 2025

**Figure 2:**
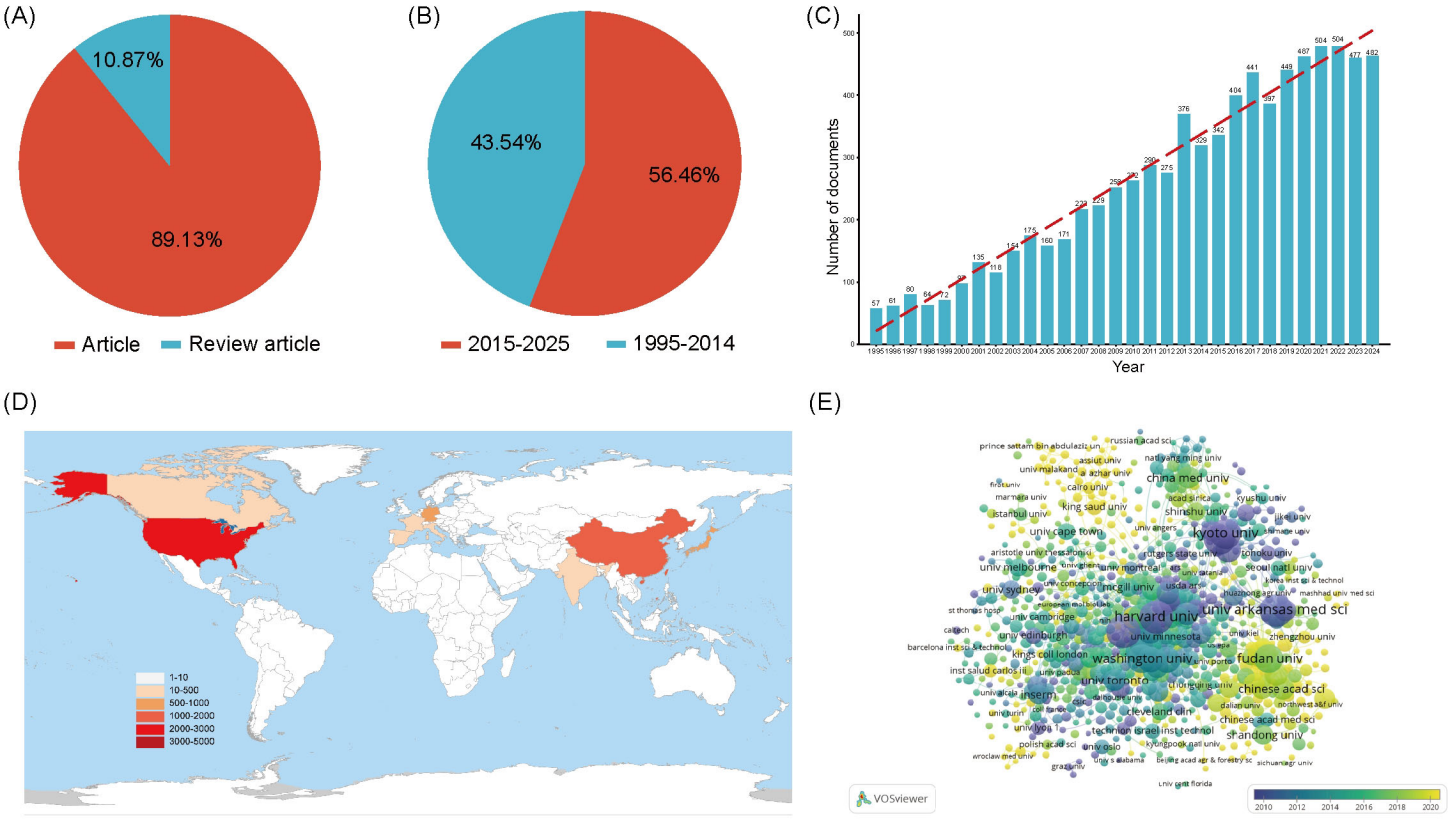
**(A)** The proportion of articles in the field of LOX. **(B)** The proportion of articles in the field of LOX over the past decade. **(C)** The number of publications in the field of LOX by year. **(D)** The top 10 most productive countries/regions in the field of LOX. **(E)** Distribution of publications from different organizations in the field of LOX. The thickness of the connecting lines between the nodes reflects the strength of the interaction. The color of the node represents the average year of publication for the country which the node represents.

By applying the search strategy TS=((“lysyl oxidase” OR “LOX” OR “LOXL1” OR “LOXL2” OR “LOXL3” OR “LOXL4” OR “lysyl oxidase like 1” OR “lysyl oxidase like 2” OR “lysyl oxidase like 3” OR “lysyl oxidase like 4”OR “Protein - lysine 6 - oxidase” OR “Copper - dependent amine oxidase”)NOT(lipoxygenase OR “lactate oxidase” OR “liquid oxygen”)) in WoSCC, Scopus, PubMed, and Embase database, a total of 9,261 LOX - related publications were identified over the past 30 years. Among these, 8,255 (89.13%) were articles, and 1,006(10.87%) were reviews (**Figure 2A**). The total number of articles in the past decade was 4,440, accounting for 56.46% of the total articles in the past 30 years (**Figure 2B**). The number of article - type publications from 1995 to 2025 showed an upward trend (**Figure 2C**). Before 2000, the annual publication count was less than 100. After 2013, there was a sharp rise in the number of relevant publications, indicating growing attention and prosperity in this field over the past 10 years.

8,255 documents in total were published by 102 countries/regions. **Figure 2D** lists the top 10 productive countries/regions for LOX publications. The United States had the most publications and citations (n = 2,944, 35.7%), followed by China (n = 1,689, 20.5%) and Japan (n = 849, 10.3%). These three countries accounted for nearly 66.5% of the total publications (**Table 2**). The 8,255 analyzed documents were affiliated with 6,489 organizations. **Table 3** shows the top 20 most productive institutions, with Harvard University and Boston University leading. Harvard University, the University of California, San Francisco, and the University of Pennsylvania had the highest citation counts (**Table 3**). **Figure 2E** depicts the overall connection strength and collaboration network among institutions.

**Table 2:** The top 10 most productive countries/regions in the field of LOX.

Rank	Country	Documents	Citations	Total link strength
1	USA	2944	173096	1623
2	China	1698	36064	495
3	Japan	849	37539	429
4	Germany	576	30399	610
5	France	369	17462	344
6	England	364	19964	458
7	Italy	298	13353	294
8	Canada	292	12751	296
9	India	292	5453	150
10	Spain	225	12823	198

**Table 3:** The top 20 most productive organizations in the field of LOX.

Rank	Organization	Country	Documents	Citations	Total link strength
1	Harvard University	USA	135	16998	264
2	Boston University	USA	133	7231	136
3	University of Arkansas for Medical Sciences	USA	128	7800	202
4	Kyoto University	Japan	110	7780	150
5	National Cardiovascular Center	Japan	106	9028	167
6	Fudan University	China	99	2869	84
7	Washington University in St. Louis	USA	98	4344	168
8	Shanghai Jiao Tong University	China	93	1952	106
9	University of Michigan	USA	82	7109	151
10	University of Tokyo	Japan	80	4276	121
11	University of California, San Diego	USA	78	4796	116
12	Johns Hopkins University	USA	75	4958	111
13	University of Pennsylvania	USA	75	10433	152
14	Baylor College of Medicine	USA	67	5721	138
15	Sun Yat-sen University	China	66	1754	83
16	University of California, San Francisco	USA	66	14282	160
17	University of California, San Francisco	USA	64	7360	125
18	Chinese Academy of Sciences	China	63	1922	77
19	Vanderbilt University	USA	63	3719	95
20	China Medical University	China	62	1557	177

### Keyword analysis of hotpots on LOX in human diseases

**Figure 3:**
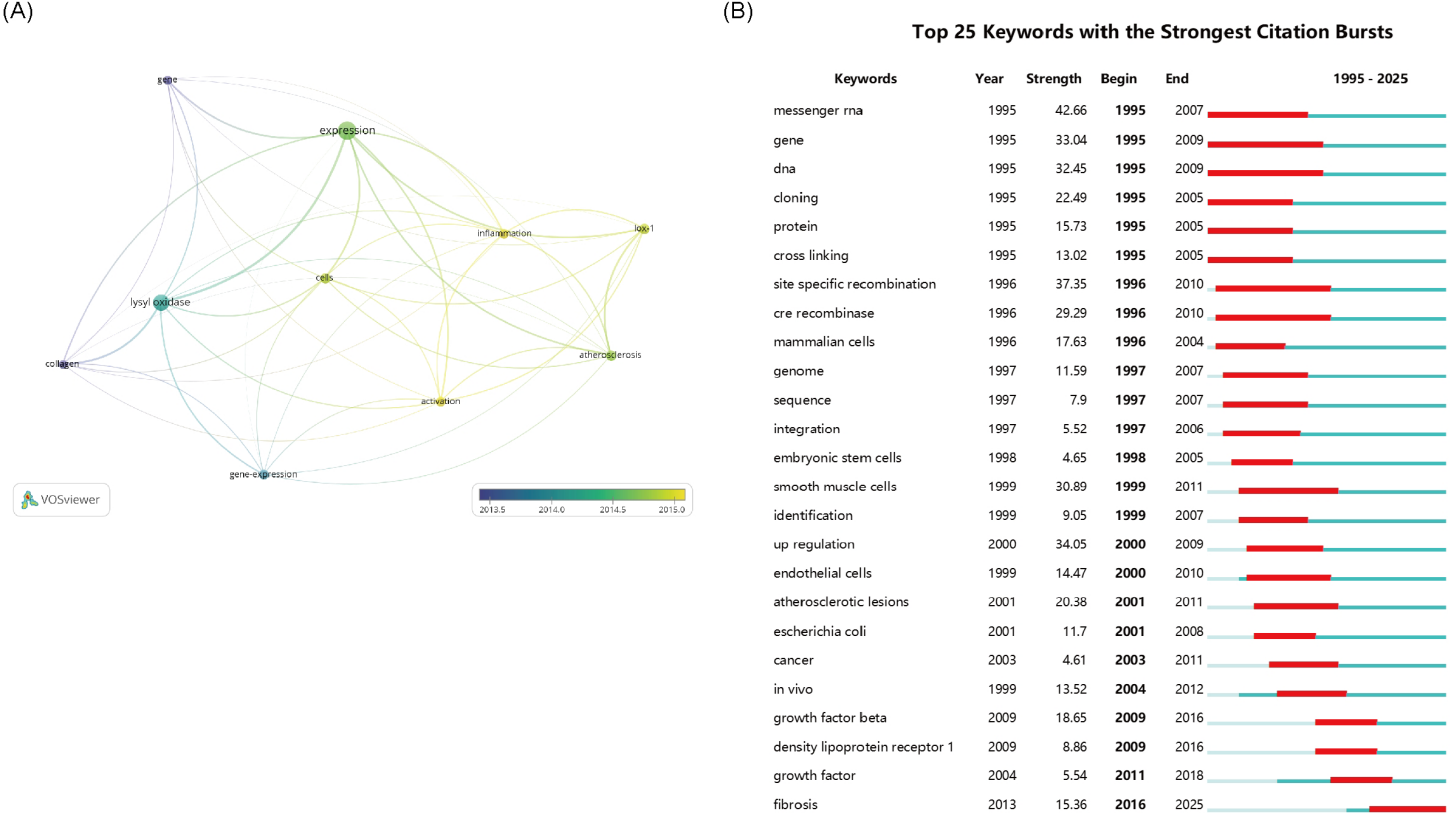
**(A)** The network visualization map of disease-related keywords. **(B)** The 25 keywords with the Strongest Citation Bursts. The thickness of the connecting lines between the nodes reflects the strength of the interaction. The color of the node represents the average year of publication for the country which the node represents.

Research themes and hotspots were identified through keyword analysis. The VOSviewer was applied to visualize the keyword network diagram for 8,255 articles. Specifically, 272 key keywords with a frequency of at least 40 were selected for visualization. **Figure 3A** shows an overlay map of the top 10 frequently occurring keywords, where the color corresponds to average publication year. Subsequently, disease - related keywords were extracted for further investigation. Clustering of these disease - related keywords resulted in 3 clusters, encompassing a total of 11 items. Among them, “cancer” and “fibrosis” appeared more than 440 times, likely representing prominent research topics. This study also presents the timeline viewer for “cancer” and “fibrosis” (**Figure 3B**).

### Analysis of literatures on LOX in fibrosis research

**Figure 4:**
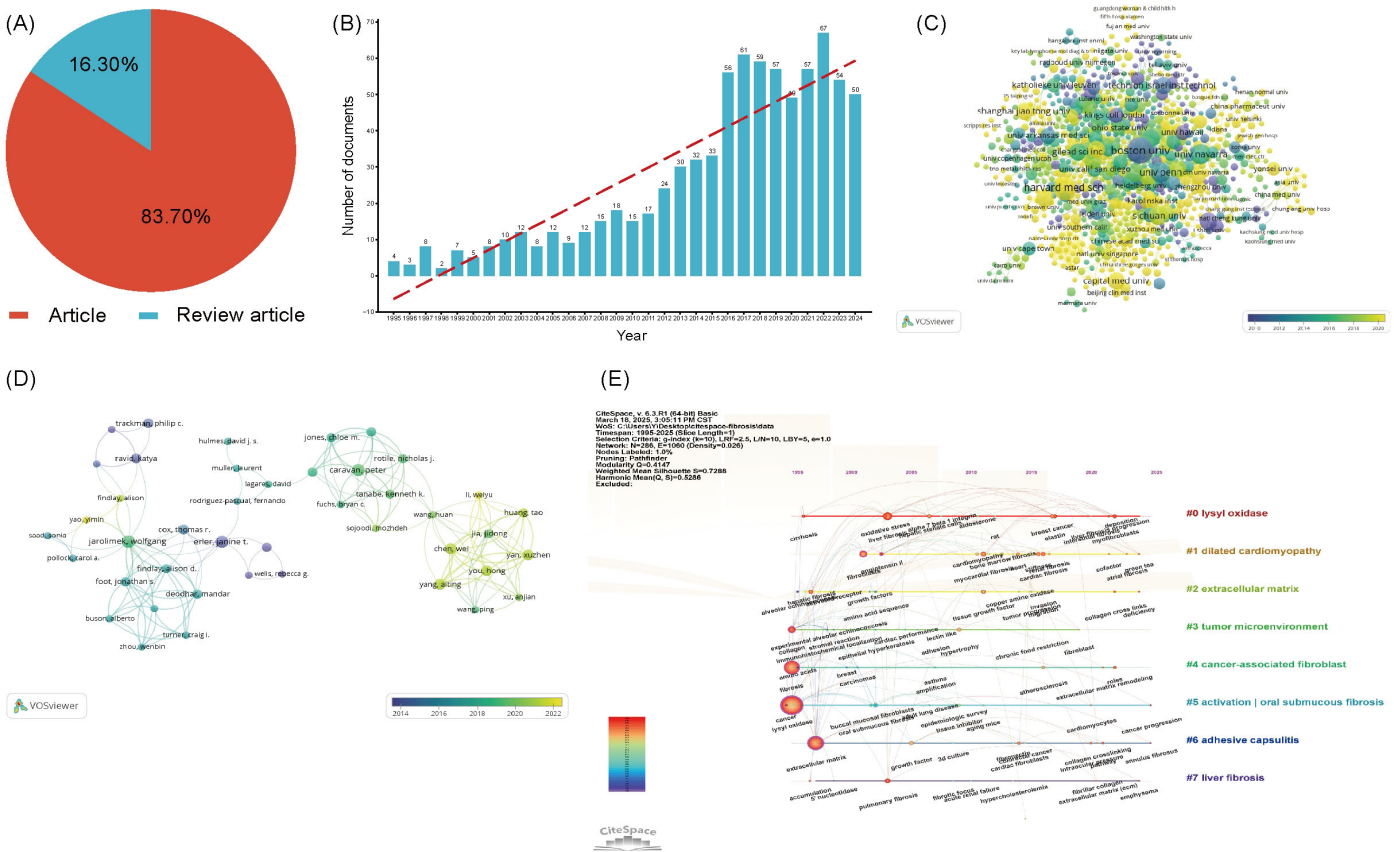
**(A)** The proportion of articles in the field of LOX associated with fibrosis research. **(B)** The number of publications in the field of LOX associated with fibrosis research by year. **(C)** Distribution of publications from different organizations in the field of LOX associated with fibrosis research. **(D)** The co-occurrence author map of co-authors in the field of LOX associated with fibrosis research. **(E)** Timeline view of keywords analysis in the field of LOX associated with fibrosis research. The thickness of the connecting lines between the nodes reflects the strength of the interaction. The color of the node represents the average year of publication for the country which the node represents.

To conduct an in - depth analysis of LOX - related literature within the realm of fibrosis research, we implemented a comprehensive search strategy. The search terms were configured as follows: TS=“lysyl oxidase” OR “LOX” OR “LOXL1” OR “LOXL2” OR “LOXL3” OR “LOXL4” OR “lysyl oxidase like 1” OR “lysyl oxidase like 2” OR “lysyl oxidase like 3” OR “lysyl oxidase like 4”OR “Protein - lysine 6 - oxidase” OR “Copper - dependent amine oxidase” AND TS=(“fibrosis” OR “fibrotic” OR “fibrogenesis”) NOT TS=(“lipoxygenase” OR “lactate oxidase” OR “liquid oxygen”)with the time span ranging from 1995 to 2025. Over these 30 years, a total of 993 LOX - related publications were identified. Among them, 831 (constituting approximately 83.7%) were original research articles, while 162 (roughly 16.3%) were review articles, as presented in **Figure 4A**. Notably, the past nine years have witnessed a remarkable upsurge in studies focusing on LOX in the context of fibrosis, as vividly illustrated in **Figure 4B**.

These 831 documents were sourced from 56 different countries and regions. The United States took the lead with 335 literatures, making up 40.3% of the total quantity, closely followed by China with 150 publications (18.1%). Together, these two countries contributed nearly 58.4% of all the relevant works, as detailed in **Table 4**. The 831 analyzed documents were affiliated with 1288 distinct organizations. Among the top 20 productive institutions, as presented in **Table 5**, Boston University (with 23 publications) and Harvard Medical School (19 publications) were particularly active. The University of Pennsylvania and the University of California, San Francisco boasted the highest citation counts, and their collaborative network is depicted in **Figure 4C**.

**Table 4:** The top 10 most productive countries/regions in the field of LOX associated with fibrosis research.

Rank	Country	Documents	Citations	Total link strength
1	USA	335	23710	195
2	China	150	3702	54
3	Germany	59	4562	78
4	Japan	54	2660	41
5	England	50	3114	72
6	France	42	2372	48
7	Spain	41	2486	39
8	Canada	37	1841	40
9	Australia	33	2103	40
10	Netherlands	26	1288	28

**Table 5:** The top 20 most productive organizations in the field of LOX associated with fibrosis research.

Rank	Organization	Country	Documents	Citations
1	Boston University	USA	23	1160
2	Harvard Medical School	USA	19	1168
3	University of Navarra	Spain	14	1210
4	University of Pennsylvania	USA	14	5251
5	Sichuan University	China	13	151
6	University of California, San Francisco	USA	12	3724
7	Massachusetts General Hospital	USA	11	468
8	National Taiwan University	Taiwan, China	11	1534
9	University of Oslo	Norway	11	412
10	Capital Medical University	China	10	178
11	Gilead Sciences, Inc.	USA	10	1074
12	Johns Hopkins University	USA	10	570
13	Medical University of South Carolina	USA	10	687
14	Pharmaxis Ltd	Australia	10	359
15	Shanghai Jiao Tong University	China	10	167
16	Technion – Israel Institute of Technology	Israel	10	1163
17	University of Kansas	USA	10	346
18	University of Michigan	USA	10	1368
19	University of Sydney	Australia	10	829
20	University of Arkansas for Medical Sciences	USA	9	316

In the past decade, 5,438 authors made contributions to LOX and fibrosis - related articles. The most productive author had 10 publications. Core contributors were defined as those with at least two publications. Employing VOSviewer, a network visualization was constructed. Authors with three or more publications were selected for further in - depth analysis, including visualization, clustering, and mapping of co - author distribution. In the fibrosis - related LOX study, 37 authors publishing five or more papers. Javier Diez ranked first with 10 publications, trailed by Wolfgang Jarolimek (8) and caravan, peter (7), as shown in **Figure 4D**. **Table 6** presents the top 20 co-cited references, with the most - cited one being from *Nature Medicine* in 2010 by Barry - Hamilton V. This reference explored LOX’s role in the tumor microenvironment, especially in metastasis and angiogenesis. **Figure 4E** showcases a keyword network for LOX research. “Lysyl oxidase” is interconnected with terms such as “dilated cardiomyopathy”, “tumor microenvironment”, and those related to fibrosis, vividly demonstrating LOX’s diverse functions across various medical conditions. Keywords like “cancer” and “extracellular matrix” stand out due to their high frequency, and the color - coded lines further reveal the complexity of the research landscape.

**Table 6:** The top 20 co-cited references in the fields of LOX associated with fibrosis research.

Rank	Co-cited reference	Author	Publication date	Journal	Citations	Total link strength
1	Allosteric inhibition of lysyl oxidase-like-2 impedes the development of a pathologic microenvironment	Barry-Hamilton V	2010	Nature Medicine	111	423
2	Lysyl oxidase-like-1 dysregulation in hypertrophic cardiomyopathy	López B	2010	American Journal of Physiology-Heart and Circulatory Physiology	87	197
3	Lysyl oxidase: properties, regulation and multiple functions in biology biology”	Kagan HM	2003	Journal of Cellular Biochemistry	85	259
4	Lysyl oxidase: properties, regulation and multiple functions in biology	Smith-Mungo LI	1998	Matrix Biology	71	180
5	Lysyl oxidase inhibition ameliorates angiotensin II-induced cardiac remodeling	Liu SB	2016	FASEB Journal	60	192
6	Lysyl oxidase-like 2 is a key mediator in pancreatic cancer progression	Ikenaga N	2017	Gut	57	220
7	Abnormal deposition of collagen around hepatocytes in Wilson’s disease is associated with hepatocyte specific expression of lysyl oxidase and lysyl oxidase like protein-2	Vadasz Z	2005	Journal of Hepatology	53	218
8	The tumour microenvironment: a novel target for cancer therapy	Barker HE	2012	Nature Reviews Cancer	50	220
9	Lysyl oxidase: an oxidative enzyme and effector of cell function	Lucero HA	2006	Cellular and Molecular Life Sciences	50	223
10	Matrix crosslinking forces tumor progression by enhancing integrin signaling	Levental KR	2009	Cell	49	169
11	Lysyl oxidases: a novel multifunctional amine oxidase family	Csiszar K	2001	Progress in Nucleic Acid Research and Molecular Biology	46	189
12	A molecular signature for metastasis in human solid cancers	Peinado H	2005	EMBO Journal	46	202
13	Targeting lysyl oxidase (LOX) overcomes chemotherapy resistance in triple negative breast cancer	Yang J	2016	Nature Communications	42	141
14	Comparative analysis of lysyl oxidase (LOX) family expression in carcinogenesis	Aumiller V	2017	Scientific Reports	40	199
15	A fluorometric assay for detection of lysyl oxidase enzyme activity in biological samples	Palamakumbura AH	2002	Analytical Biochemistry	40	155
16	The hypoxic cancer secretome induces pre-metastatic bone lesions through lysyl oxidase	Cox TR	2013	Cancer Research	39	133
17	Hypoxia-induced lysyl oxidase is a critical mediator of bone marrow cell recruitment to the neovasculature	Erler JT	2009	Cancer Cell	39	141
18	The expression of lysyl oxidase in oral squamous cell carcinoma	Trivedy C	1999	Journal of Oral Pathology and Medicine	38	36
19	Regulation of lysyl oxidase activity in cultured bovine aortic smooth muscle cells	Kagan HM	1991	American Journal of Respiratory Cell and Molecular Biology	37	69
20	Lysyl Oxidase Is Essential for Normal Development and Function of the Respiratory System and for the Integrity of Elastic and Collagen Fibers in Various Tissues	Mäki JM	2002	Circulation	37	150

### Analysis of literatures on LOX in cancer research

**Figure 5:**
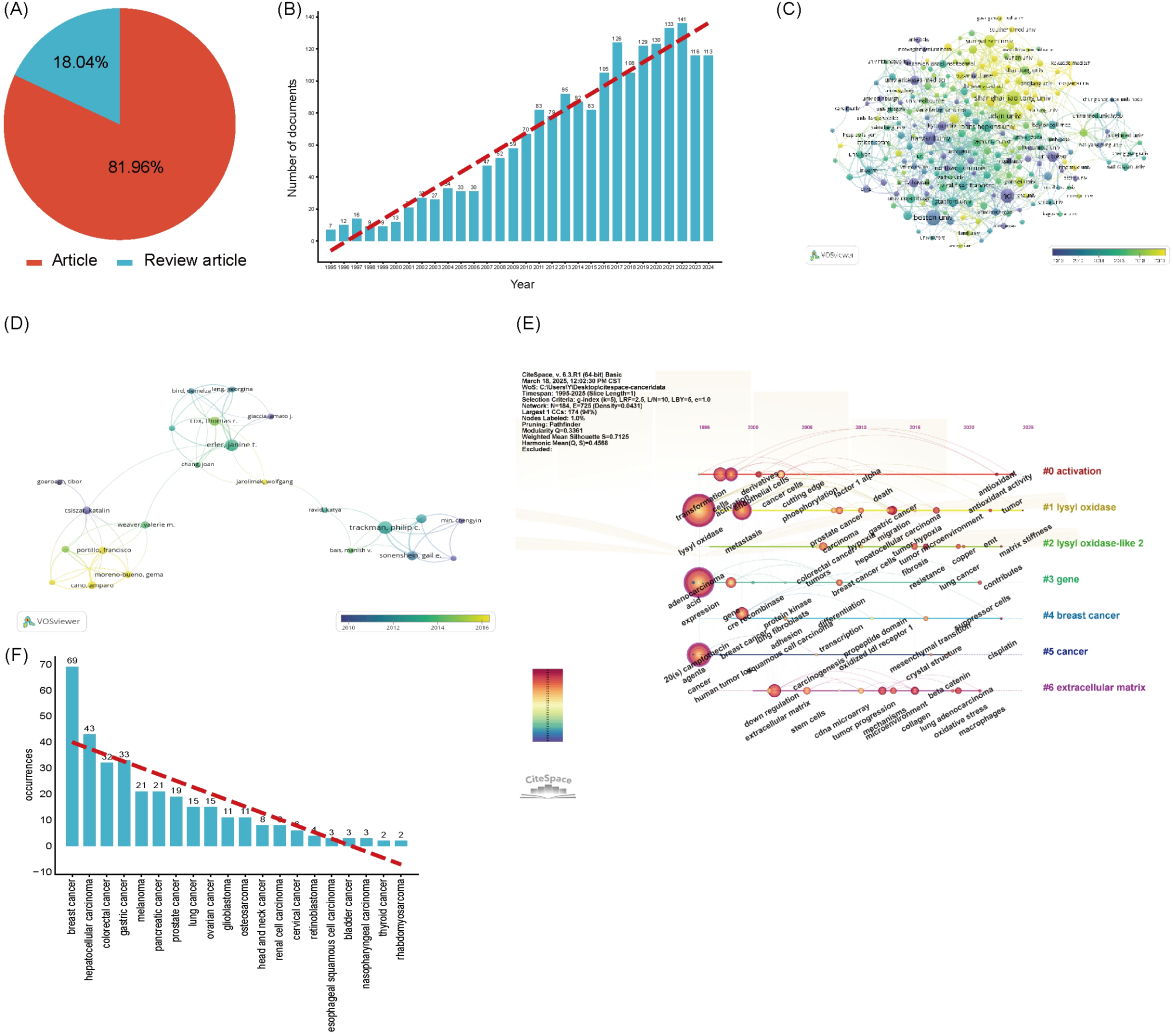
**(A)** The proportion of articles in the field of LOX associated with cancer research. **(B)** The number of publications in the field of LOX associated with cancer research by year. **(C)** Distribution of publications from different organizations in the field of LOX associated with cancer research. **(D)** Timeline view of keywords analysis in the field of LOX associated with cancer research. **(E)** The co-occurrence author map of co-authors in the field of LOX associated with cancer research. **(F)** The recurrence rate of cancer types. The thickness of the connecting lines between the nodes reflects the strength of the interaction. The color of the node represents the average year of publication for the country which the node represents.

Our search strategy for LOX in cancer research was formulated as: TS=“lysyl oxidase” OR “LOX” OR “LOXL1” OR “LOXL2” OR “LOXL3” OR “LOXL4” OR “lysyl oxidase like 1” OR “lysyl oxidase like 2” OR “lysyl oxidase like 3” OR “lysyl oxidase like 4”OR “Protein - lysine 6 - oxidase” OR “Copper - dependent amine oxidase”AND TS=(“cancer” OR “tumor” OR “neoplasm” OR “malignancy” OR “carcinoma”) NOT TS=(“lipoxygenase” OR “lactate oxidase” OR “liquid oxygen”)from 1995 to 2025. In the past 30 years, 2490 LOX - related publications in the cancer domain were unearthed. Among these, 2,041 (8%) were articles, and 449 (18%) were review articles, as shown in **Figure 5A**. The number of such publications has been steadily increasing. In the recent nine years, the annual output was approximately ten - fold that of 1996, as depicted in **Figure 5B**. These 2,041 documents originated from 78 countries and regions. The United States had the greatest number of publications (n = 704), followed by China (n = 519) and Japan (n = 175), together accounting for 68.5% of the total, as shown in **Table 7**. The 2027 analyzed documents were associated with 2,486 organizations. Among the top 20 productive academic institutions in **Table 8**, Shanghai Jiao Tong University (44), and Fudan University (42) were at the forefront. The University of California, San Francisco had the highest citation count, as manifested in the collaboration network presented in **Figure 5C**.

**Table 7:** The top 10 most productive countries/regions in the field of LOX associated with cancer research.

Rank	Country	Documents	Citations	Total link strength
1	USA	701	52026	394
2	China	506	13145	135
3	Japan	174	8375	83
4	Germany	105	4509	111
5	England	97	7200	127
6	France	87	5791	78
7	India	81	1754	28
8	South korea	79	3073	29
9	Italy	62	2730	49
10	Canada	54	3871	55

**Table 8:** The top 20 most productive organizations in the field of LOX associated with cancer research.

Rank	Organization	Country	Documents	Citations	Total link strength
1	Boston University	USA	48	1904	19
2	Shanghai Jiao Tong University	China	44	1191	21
3	Fudan University	China	42	1550	20
4	National Cancer Institute	USA	41	2158	16
5	Harvard University	USA	35	4355	26
6	Sun Yat-sen University	China	32	745	22
7	Johns Hopkins University	USA	26	2892	12
8	The University of Tokyo	Japan	24	1400	6
9	Stanford University	USA	22	5099	21
10	Harvard Medical School	USA	20	545	16
11	Kyoto University	Japan	20	1440	6
12	University of Arkansas for Medical Sciences	USA	20	1654	7
13	University of California, San Francisco	USA	20	8626	25
14	University of Hawaii	USA	20	1800	6
15	The University of Texas MD Anderson Cancer Center	USA	20	1634	22
16	Northwestern University	USA	19	1066	23
17	University of Copenhagen	Denmark	19	2349	13
18	Yonsei University	South Korea	19	544	10
19	Central South University	China	18	298	15
20	Institut National de la Santé et de la Recherche Médicale	France	18	1022	16

Over the past decade, 13,219 authors published on LOX and cancer. Using VOSviewer, a network visualization was generated. Authors with four or more publications were selected for further analysis. In the cancer - related LOX study, 137 authors were involved. Philip C. Trackman had the most publications (25), followed by Janine T. Erler (22) and Thomas R. Cox (15). Their co - authorship map is presented in **Figure 5D**. **Table 9** enumerates the top 20 co - cited references. The reference with the highest citation count was from a 2006 publication in Nature, authored by Janine T. Erler. This study delved into LOX’s role in tumor metastasis, especially its ECM - regulating mechanism for tumor invasion. **Figure 5E**’s keyword visualization displays nodes such as “activation”, “lysyl oxidase”, “lysyl oxidase - 2”, “gene”, “breast cancer”, “cancer”, and “extracellular matrix” connected by lines, highlighting the intricate relationships within LOX research. It clearly indicates that LOX research is closely intertwined with cancer, particularly breast cancer, the extracellular matrix, biological activation, and genetics.

**Table 9:** The top 20 co-cited references in the fields of LOX associated with cancer research.

Rank	Co-cited reference	Author	Publication date	Journal	Citations	Total link strength
1	Lysyl oxidase-related protein-1 promotes tumor fibrosis and progression *in vivo*	Erler JT	2006	Nature	330	1831
2	Lysyl oxidase-like 2 (LOXL2) modulates tumor-associated macrophage polarization and promotes breast cancer metastasis	Erler JT	2009	Cancer Cell	210	1189
3	Tumor-secreted LOXL2 activates fibroblasts through FAK signaling	Kagan HM	2003	Journal of Cellular Biochemistry	178	1069
4	The tumour microenvironment: a novel target for cancer therapy	Levental KR	2009	Cell	177	1147
5	Allosteric inhibition of lysyl oxidase-like-2 impedes the development of a pathologic microenvironment	Barker HE	2012	Nature Reviews Cancer	175	1049
6	Chromosomal localization of the lysyl oxidase gene (LOX) in mouse and human	Kirschmann DA	2002	Cancer Research	155	1147
7	The lysyl oxidase-like 2 enzyme regulates tumor angiogenesis and is a potential therapeutic target	Peinado H	2005	EMBO Journal	129	1017
8	Lysyl oxidases: a novel multifunctional amine oxidase family	Barry-Hamilton V	2010	Nature Medicine	126	919
9	Hypoxia-induced lysyl oxidase is a critical mediator of bone marrow cell recruitment to form the pre-metastatic niche	Lucero HA	2006	Cellular and Molecular Life Sciences	110	755
10	Lysyl oxidase is essential for hypoxia-induced metastasis	Csiszar K	2001	Progress in Nucleic Acid Research and Molecular Biology	111	786
11	Lysyl oxidase: properties, specificity, and biological roles inside and outside of the cell	Payne SL	2005	Cancer Research	101	785
12	A molecular role for lysyl oxidase in breast cancer invasion	Payne SL	2007	Journal of Cellular Biochemistry	98	769
13	Matrix crosslinking forces tumor progression by enhancing integrin signaling	Smith-Mungo LI	1998	Matrix Biology	90	556
14	Lysyl oxidase: an oxidative enzyme and effector of cell function	Cox TR	2013	Cancer Research	83	533
15	Lysyl oxidase regulates breast cancer cell migration and adhesion through a hydrogen peroxide-mediated mechanism	Peinado H	2008	Cancer Research	83	709
16	Lysyl oxidase regulates tumor cell survival through Akt-dependent and -independent mechanisms	Baker AM	2011	Journal of the National Cancer Institute	79	604
17	Lysyl oxidase-like 2 as a new regulator of epithelial-mesenchymal transition	Barker HE	2011	Cancer Research	77	617
18	LOXL2-mediated matrix remodeling in metastasis and mammary gland involution	Peng L	2009	Carcinogenesis	77	650
19	Lysyl oxidase activates cancer stromal cells and promotes gastric cancer progression	Akiri G	2003	Cancer Research	76	566
20	Lysyl oxidase: properties, regulation, and multiple functions in biology	Contente S	1990	Science	72	445

**Figure 5F** is a bar chart illustrating the recurrence occurrences of various cancer types. The x - axis lists different cancer types, ranging from breast cancer to rhabdomyosarcoma. The y - axis represents the number of recurrences. Blue bars are used to depict the recurrence counts for each cancer. Breast cancer has the highest number of recurrences, reaching 69, while rhabdomyosarcoma has the lowest, with only 2 occurrences. A red dashed line is overlaid on the chart, which shows a downward trend, indicating that the recurrence numbers generally decrease from one cancer type to another. This chart effectively presents the differences and trends in recurrence frequencies among these cancers. This research on LOX encompasses the top ten most common tumors globally, with studies on lung cancer (15 documents), breast cancer (69 documents), colorectal cancer (32 documents), prostate cancer (19 documents), stomach cancer (33 documents), liver cancer (43 documents), cervical cancer (6 documents), thyroid cancer (2 documents), and bladder cancer (3 documents), but excludes research on esophageal cancer.

## Discussion

In one of their other studies, it was revealed that LOX, secreted by hypoxic breast tumor cells, accumulates at pre - metastatic sites. It crosslinks collagen IV in the basement membrane, playing a pivotal role in the recruitment of CD11b+ myeloid cells (36). These CD11b+ cells bind to the crosslinked collagen IV and produce matrix metalloproteinase - 2, which cleaves collagen, thereby facilitating the invasion and recruitment of bone marrow - derived cells (BMDCs) and metastasizing tumor cells (46, 47).Regarding human colorectal carcinoma cell lines, the induction of LOX augmented HIF - 1 expression, whereas LOX silencing led to a reduction. Mechanistic investigations disclosed that LOX activated the PI3K (phosphoinositide 3 - kinase) - Akt signaling pathway, which, in turn, upregulated the synthesis of HIF - 1α protein in a manner contingent on LOX - mediated hydrogen peroxide production (48–50).

LOX proteins, especially LOXL2, are enzymes involved in the cross - linking of collagen fibers, thus playing a critical role in the stabilization of the extracellular matrix (ECM) (51–53). In cancer, dysregulation of these enzymes has been implicated in tumor progression and metastasis (42). The ECM serves as both a structural scaffold and a signaling hub, and its abnormal remodeling by LOX proteins contributes to the mechanical properties of tumors, facilitating invasion and metastasis. Emerging evidence suggests that LOX activity is regulated by various signaling pathways, including TGF - β and Wnt/β - catenin, which are frequently dysregulated in cancer (30, 54–56). Understanding the molecular mechanisms underlying LOX function could offer novel insights into tumor biology and therapeutic strategies (35, 57, 58).

The expression of LOX proteins has been shown to correlate with poor clinical outcomes in several cancers, such as breast, prostate, and ovarian cancers (59–61). This is largely attributable to their role in enhancing tumor stiffness, which promotes cancer cell survival, proliferation, and migration. Additionally, LOX enzymes may contribute to the maintenance of cancer stem cell properties by altering the ECM niche, thereby supporting tumor recurrence and resistance to therapy (28, 62, 63). Furthermore, LOX proteins are known to interact with other components of the tumor microenvironment, including immune cells, further complicating their role in cancer progression. These findings underscore the necessity for a comprehensive understanding of LOX - mediated ECM remodeling in cancer pathogenesis (64–67).

The outcomes of the mapping in **Figure 4E** suggest promising future research directions. These include delving into the interactions between LOX and the extracellular matrix during cancer metastasis and invasion, as well as exploring related gene regulatory pathways (68–72). Such research could potentially unlock novel approaches for diagnosing and treating diseases by targeting LOX (73–75). Inhibiting Lox enzymes has emerged as a promising therapeutic approach to combat cancer metastasis (76–81). Preclinical studies have demonstrated that targeting LOXL2 can reduce tumor stiffness, impair cancer cell invasion, and sensitize tumors to chemotherapy. Currently, several inhibitors of LOX enzymes are undergoing preclinical development and early - stage clinical trials (82, 83). These inhibitors aim to disrupt the cross - linking of collagen fibers, thereby reducing tumor stiffness and limiting cancer cell dissemination (84–90). Additionally, understanding the regulatory mechanisms of LOX expression could lead to novel therapeutic approaches, such as targeting upstream signaling pathways or combining LOX inhibition with immunotherapy to enhance anti - tumor immune responses (91–93). However, further research is needed to optimize these strategies and address potential off - target effects (94–96).

Lysyl oxidase (LOX) fulfills a critical function in vascular fibrosis and extracellular matrix (ECM) remodeling by catalyzing the oxidation of lysine residues in collagen and elastin, thereby facilitating ECM cross-linking and contributing to vascular wall stiffening (22). It orchestrates the phenotypic transition of vascular smooth muscle cells (VSMCs) from a contractile to a synthetic phenotype via the transforming growth factor-β (TGF-β)/Smad signaling cascade, which accelerates fibrotic processes and diminishes vascular compliance (97). LOX exacerbates vascular inflammation and endothelial dysfunction by activating the nuclear factor-κB (NF-κB) pathway, culminating in the secretion of pro-inflammatory cytokines, including interleukin-6 (IL-6) and tumor necrosis factor-α (TNF-α) (98). Additionally, LOX contributes to oxidative stress-induced damage to vascular endothelial cells, compromising endothelial barrier integrity and initiating atherosclerotic plaque formation (99). It enhances matrix metalloproteinases (MMPs) activity, leading to collagen degradation in the fibrous cap, which impacts plaque stability and the risk of acute cardiovascular events (100). LOX may also polarize macrophages toward a pro-inflammatory M1 phenotype, augmenting lipid phagocytosis and foam cell formation, while suppressing polarization to the anti-inflammatory M2 phenotype, thereby exacerbating intra-plaque inflammation and lipid accumulation (101). LOX interacts with lipid metabolism by promoting LDL oxidation to form oxidized LDL (ox-LDL) and can inhibit ATP-binding cassette transporters, obstructing cholesterol reverse transport from foam cells to high-density lipoprotein (HDL) (102). Therapeutic interventions targeting LOX, such as β-aminopropionitrile (BAPN) and LOXL2-specific inhibitors like GSK2878163, have demonstrated efficacy in mitigating ECM cross-linking and decelerating atherosclerosis progression in animal models (20). Early studies in chickens and pigs exhibited variable effects of dietary interventions on LOX activity, whereas in rabbits with advanced atherosclerosis and ApoE-deficient mice on a high-fat diet, LOX activity was elevated, and BAPN treatment ameliorated atherosclerosis (103). LOX expression is also implicated in human plaque stability. Its involvement in collagen cross-linking and its regulation by factors such as hypoxia and cytokines highlight its pivotal role in plaque stability, necessitating further investigation into its contribution to plaque instability (7, 20, 104)

**Figure 6:**
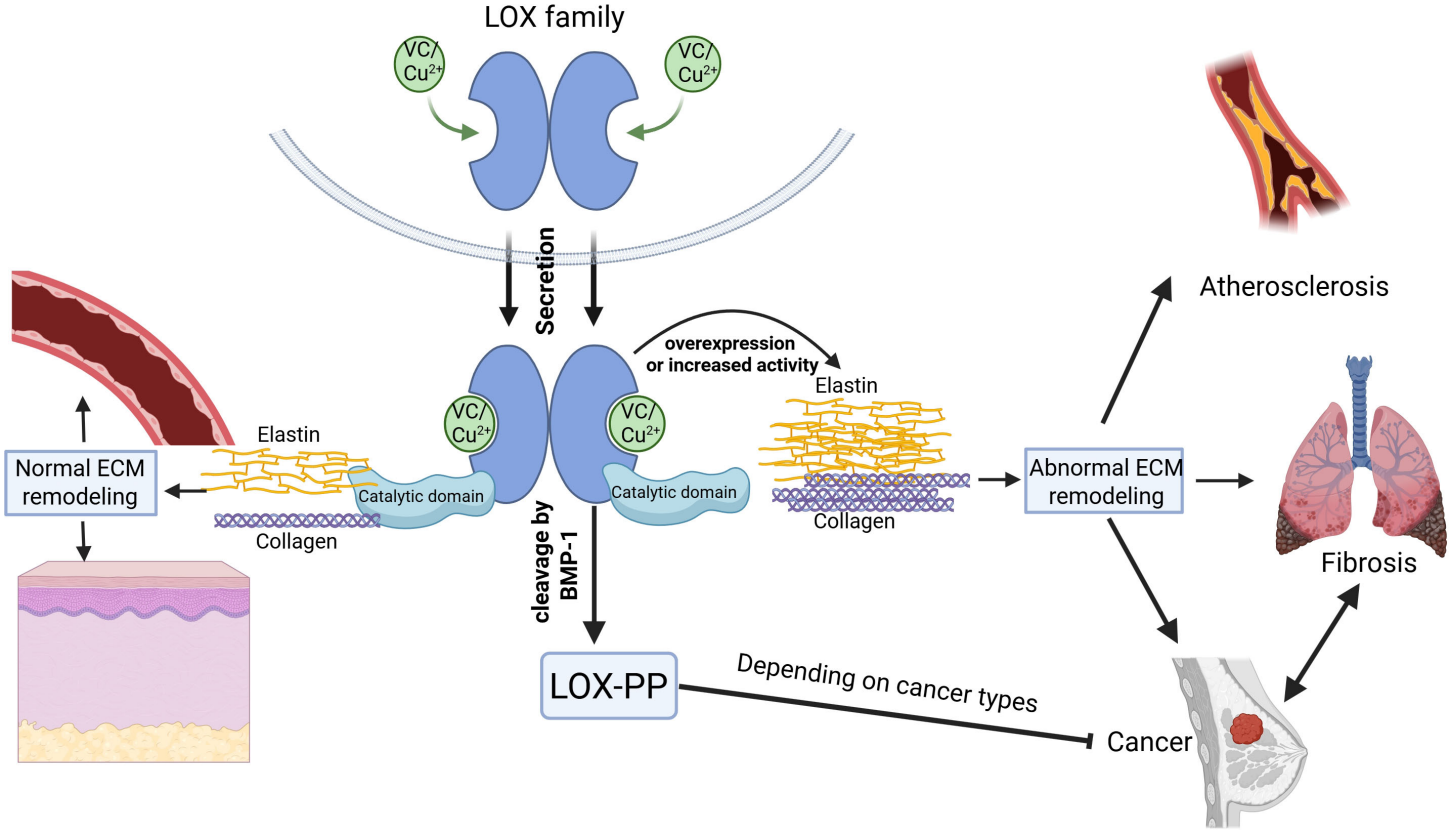
The role of the LOX family in extracellular matrix (ECM) remodeling. The LOX family proteins, dependent on vitamin C and copper ions for their activity, contain both a catalytic domain and a propeptide domain. They play a crucial role in normal ECM remodeling involving elastin and collagen under physiological conditions. Overexpression or increased activity of LOX proteins, however, results in abnormal ECM remodeling linked to pathologies such as atherosclerosis, organ fibrosis, and various cancers. The LOX family acts as a key regulator in the transition between fibrosis and cancer. When LOX is secreted outside the cell, the N - terminus of the protein is cleaved by BMP – 1, forming the LOX propeptide (LOX - PP) and the catalytic domain of LOX protein. The catalytic domain of LOX proteins can promote cancer progression, whereas the LOX-PP has been shown to suppress cancer growth in certain types of cancer. WANG, Z. (2025) https://BioRender.com/2pa62d9.

Fibrosis and cancer intersect through the lysyl oxidase (LOX) enzyme, both sharing a disrupted extracellular matrix (ECM) microenvironment. LOX acts as a pivotal mediator in this context. Current fibrosis research has emphasized a detailed analysis of LOX isoform expression, uncovering its overexpression in lung, liver, heart, skin fibrosis, and hypertension. Barry-Hamilton discovered that LOXL2 drives the pathologic microenvironment of cancer and fibrotic diseases in lung and liver, with elevated levels in diseased stroma. AB0023, an inhibitory antibody targeting LOXL2, effectively reduced disease markers in both cancer and fibrosis models (95). Inflammation-driven conditions such as hepatitis not only foster fibrosis but also contribute to carcinogenic mutations, with LOX sustaining a pro-inflammatory and ECM rigidifying niche, thereby perpetuating an “inflammation-fibrosis-cancer” continuum (105, 106). Conversely, in fibrosis linked to cancer, LOX and transforming growth factor-β (TGF-β)-activated myofibroblasts secrete growth factors like HGF and FGF, which promote tumor cell viability and angiogenesis LOX’s enzymatic role extends to myelofibrosis, influencing megakaryocyte differentiation and platelet formation, and is critical for PDGF signaling and cell proliferation (107, 108). In a GATA-1-deficient myelofibrosis model, LOX overexpression drives ECM buildup and marrow fibrosis, which BAPN can mitigate. Elevated LOX activity in the marrow significantly contributes to myelofibrosis, with human myeloproliferative neoplasms showing heightened expression of several LOX isoforms (109). In mesothelioma, the build-up of asbestos in the pleural cavity causes prolonged inflammation, which prompts the malignant transformation of mesothelial cells and leads to fibrosis (110, 111). Therapeutically, LOX inhibition could concurrently benefit fibrosis and cancer treatment, such as LOXL2 inhibitors reducing ECM stiffness in fibrotic tumors to improve drug efficacy. Antifibrotic approaches include non-selective LOX inhibitors like BAPN and selective LOXL2 inhibitors, as well as MSC therapy, which can decrease LOX expression in liver injury models (3). The relationship between LOX family and extracellular matrix remodeling among various diseases is graphically represented in **Figure 6**.

## Conclusion

This bibliometric analysis of LOX research from 1995 to 2025 identified 9,261 related publications. Keyword analysis showed “cancer” and “fibrosis” as key hotspots. LOX’s diverse functions and its links to cancer and fibrosis. This study provides an overview of LOX research trends, hotspots, and collaborations, guiding future research on LOX in diseases and therapies.

## Future perspectives

Through our analysis of original research on LOX, we found that breast cancer accounts for the largest number of LOX-related studies. Therefore, we conducted a further in-depth analysis of LOX research in breast cancer. The results showed that: in breast cancer, the expression of LOXL1 is significantly upregulated, while that of LOX, LOXL2, and LOXL3 remains unchanged. Conversely, LOXL4 shows significant downregulation. Among these, only elevated LOXL2 expression demonstrates a strong correlation with a shortened disease - free survival duration in breast cancer patients, highlighting its potential as a critical biomarker for prognosis (59). The promotion of breast cancer progression by LOX family molecules is primarily mediated through several key mechanisms. Firstly, collagen cross - linking occurs, which contributes to the alteration of the extracellular matrix microenvironment (112). Secondly, the induction of epithelial - mesenchymal transition (EMT) is achieved by downregulating the expression of the E - cadherin epithelial marker and simultaneously upregulating the transcription of TWIST, thus facilitating the acquisition of a mesenchymal phenotype by cancer cells (113). Thirdly, the activation of the FAK/Src signalling pathway plays a pivotal role, triggering a cascade of intracellular events that enhance cell motility and invasiveness (28). Finally, the establishment of a premetastatic niche involving bone marrow-derived cells (BMDCs) is accomplished via collagen restructuring, providing a supportive microenvironment for the dissemination and colonization of cancer cells (114). As we look to the future of breast cancer research and treatment, the exploration of LOX family proteins holds immense promise. Despite the significant strides made in breast cancer diagnosis and prognosis with medical advancements and biomarker discoveries, distant metastasis remains a formidable challenge to patient survival.

The emerging role of LOX family proteins in promoting breast cancer, from regulating initiation and spread to mediating intracellular EMT remodelling, positions them as pivotal players in the disease process. Their potential as biomarkers for early detection, disease staging, and evaluating chemotherapy effectiveness, along with their candidacy as targets for metastasis prevention, has already been recognized. The development of various inhibitors, though showing promise, also underscores the need for a more refined therapeutic approach considering their differential selectivity for LOX family enzymes.

Moving forward, novel drug therapies targeting LOX family proteins are likely to be at the forefront of personalized and precise treatment strategies for breast cancer patients. An especially exciting frontier lies in investigating the role of LOX family proteins in the tumour immune microenvironment. Unravelling the regulatory mechanisms of these proteins on the breast cancer immune microenvironment and exploring the synergistic effects with immunotherapy could open new vistas in treatment.

To fully harness the potential of LOX family proteins in breast cancer management, several key research areas demand attention. There is an urgent need to bridge the knowledge gap regarding the oncogenic effects of LOXL1 and LOXL3 in breast cancer through in - depth fundamental and clinical studies. Additionally, while downstream targets of LOX family proteins have been the focus of existing research, understanding their upstream gene regulation and degradation pathways is essential. The use of gene - edited cells and mouse models can facilitate such investigations.

Identifying effective biomarkers, clarifying substrate - binding mechanisms, and understanding the molecular intricacies of LOX family proteins in breast cancer are crucial steps. Determining the optimal time for intervention to maximize treatment efficacy is another promising avenue. Given the pre - clinical success of LOX - targeting drugs in breast cancer and their efficacy in other cancer types’ clinical trials, further research into miR - mediated mechanisms of LOX family member activity could illuminate the tumour microenvironment’s molecular landscape and lead to innovative clinical therapies.

## Limitations

Despite the significant contributions of existing research on LOX family, several limitations should be noted. First, potential omissions may arise due to inappropriate synonyms or abbreviations, as the keyword-based search strategy might have missed or mis-included relevant studies using alternative terminology introducing risks of incomplete retrieval due to semantic variability, particularly in fields with evolving nomenclature. Second, database-specific coverage limitations exist: although representative databases (e.g., PubMed, Web of Science) were systematically searched, other databases (e.g., CINAHL, BIOSIS Previews) and non-English/regional databases (e.g., CNKI) may have led to unintentional gaps, potentially missing valuable data on LOX in fibrosis or cancer. Third, temporal gaps in database updates constrain timeliness: even with a standardized search date, newly published studies—especially in rapidly advancing fields like precision oncology—may not yet be indexed, risking omission of breakthroughs in LOX-targeted therapies published shortly before the search date and affecting the review’s currency. Finally, the inability to capture post-submission retractions poses a challenge: studies retracted after manuscript submission could not be excluded, as retraction notices often lag behind original publications. While study integrity was verified at the time of review, unforeseen post-submission retractions introduce a minor risk of outdated or invalid data persisting, a common limitation in cross-sectional reviews that underscores the importance of ongoing critical evaluation in scientific discourse.

## Data Availability

The original contributions presented in the study are included in the article/supplementary material. Further inquiries can be directed to the corresponding author.
